# Empyema necessitans as a rare manifestation of *Staphylococcus aureus*

**DOI:** 10.1002/ccr3.8697

**Published:** 2024-04-01

**Authors:** Nazanin Zeinali Nezhad, Aazam Gholami Shahrebabak, Amirhossein Shahpar

**Affiliations:** ^1^ Physiology Research Center, Institute of Neuropharmacology Kerman University of Medical Sciences Kerman Iran; ^2^ Department of Medical Education, Medical Education Research Center Kerman University of Medical Sciences Kerman Iran; ^3^ Gastrointestinal Research Center Kerman University of Medical Sciences Kerman Iran

**Keywords:** empyema necessitans, pediatric, *Staphylococcus*, treatment

## Abstract

Empyema necessitans (EN) is a rare complication of empyema, in which pus accumulates within the pleural space and spreads through the chest wall, leading to the formation of a subcutaneous abscess. This condition presents significant diagnostic and therapeutic challenges due to its rarity and potential for serious complications. Here, we present the case of an 8‐year‐old boy with a history of parapneumonic effusion that was incompletely treated due to a lack of fibrinolytic agent injection. He presented with fever, chills, a productive cough, and left‐sided chest pain with yellowish purulent secretions from the left chest wall. The patient was diagnosed with EN caused by *Staphylococcus aureus*, which has occurred due to inadequate treatment and the lack of administration of a fibrinolytic agent injection for the patient. He was treated with broad‐spectrum antibiotics, video‐assisted thoracic surgery, and a chest tube for complete drainage. The patient showed a smooth and uneventful recovery, highlighting the importance of early diagnosis and prompt treatment of EN to avoid further complications. This case report aims to increase awareness among clinicians about the importance of early recognition and appropriate management of EN to improve patient outcomes.

## 1 INTRODUCTION

Empyema necessitans (EN), also known as empyema necessitates, is an infrequent pathological process that may arise when an empyema penetrates and breaches the parietal pleural membrane, subsequently extending beyond the pleural space and following the path of least resistance through the adjacent soft tissues.^1^ It typically arises as a secondary complication of an underlying pneumonia or parapneumonic effusion, although it may also occur as a consequence of an infection at alternative sites.^2^ Other potential etiologies of EN comprise chest trauma or surgical procedures, albeit rare, as well as foreign body aspiration.^3^ EN may also occur in immunocompromised individuals, such as those receiving immunosuppressive therapy or with underlying diseases such as HIV or cancer.^4^ EN may manifest with various clinical symptoms, including but not limited to local pain, cough, dyspnea, fever, and weight loss.^5^ The symptoms of EN can be managed with a range of treatment options, including the administration of antibiotics, tube drainage, and decortication, which can effectively eliminate the cavity and restore optimal pulmonary function.^6^ In this report, we present a case of pediatric Staphylococcus‐associated EN, which was characterized by the presence of purulent drainage from the chest wall. The patient received treatment through open drainage by video‐assisted thoracic surgery (VATS), a tube thoracotomy, and a course of antibiotics.

## 2 CASE REPORT

An eight‐year‐old boy patient, who had previously been admitted to Zahedan Hospital due to parapneumonic effusion 1 month prior. After tapping the plural fluid, the specimen was sent for analysis and culture, which is summarized in Table 1.

**TABLE 1:** Plural effusion analysis at the first admission.

Appearance	Turbid (pus)
Protein	4 mg/dL
Glucose	27 mg/dL
LDH	1300 U/L
Plural fluid PH	6.7
Microscopic evaluation	PMN predominance
Culture	*Staphylococcus aureus*

**FIGURE 1:**
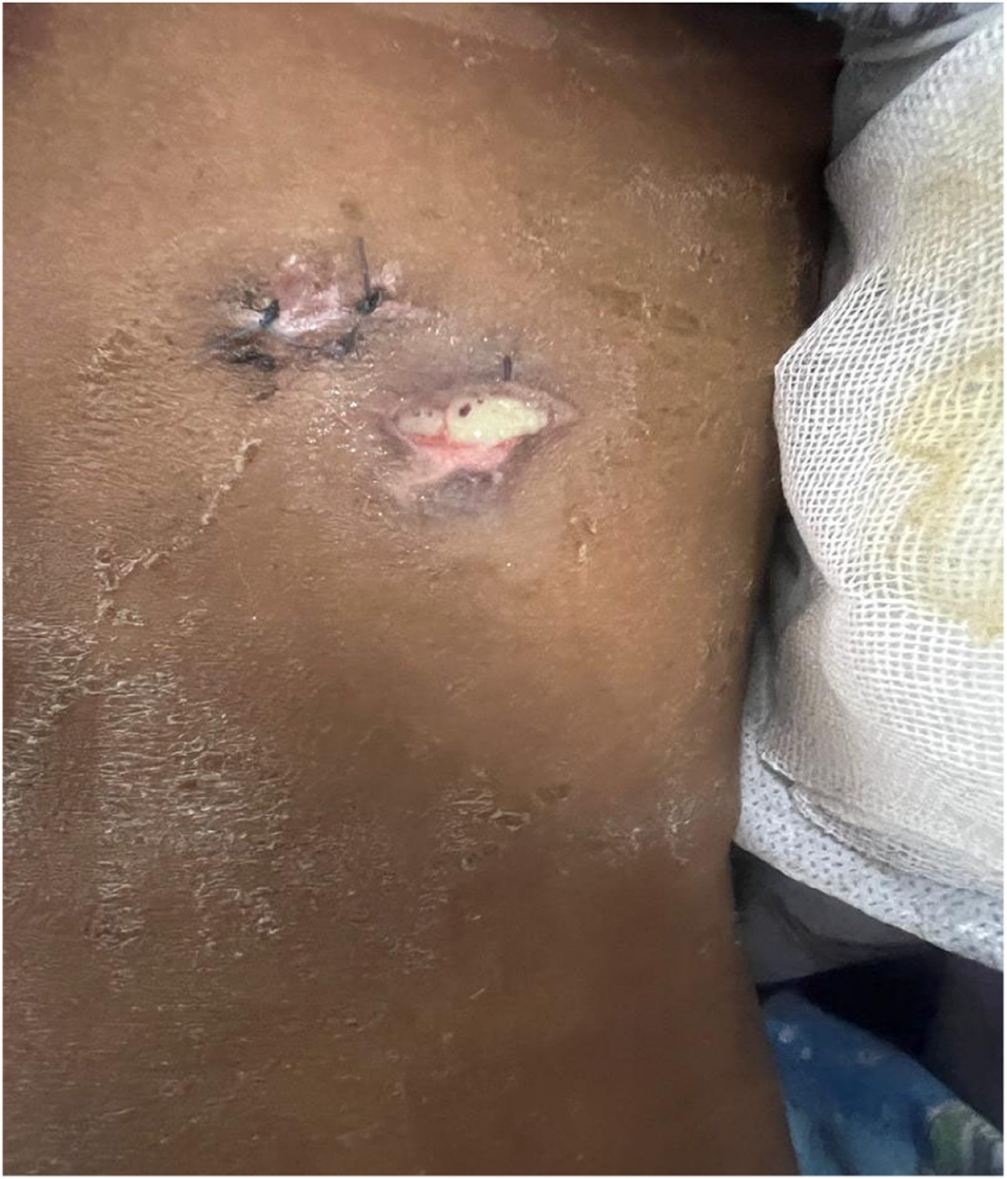
The yellowish secretions from the patient's left chest wall.

The patient received incomplete treatment involving a chest tube, antibiotics, and lacked the administration of a fibrinolytic agent injection. After on month, he presented with symptoms of fever, chills, and a productive cough, as well as pain in the left arm and left‐sided chest. He also complained of yellowish, purulent secretions from the left chest wall within the 2 weeks before admission (Figure 1).

On examination, he was ill, tachypneic (30 breaths per minute), and febrile, with a corrected axillary temperature of 38.9°C. His oxygen saturation in room air was 90%, and his blood pressure was within normal limits. There were multiple enlarged, tender supraclavicular and axillary lymph nodes observed on the left side. The lymph nodes measured approximately 0.5 cm and were found to be mobile with a firm consistency.

On the chest examination, a visible scar from the previous chest tube was observed on the left side of the chest. In proximity to the chest tube scar, a fistula measuring 2 cm was detected. The tract was found to be actively discharging yellowish, purulent drainage. In chest auscultation, breath sounds were reduced in the left lower zone of the lung with few crepitations. Other systemic examinations were found to be normal.

The patient visited a physician with the mentioned complaints and was prescribed oral therapy consisting of clindamycin capsules 150 mg four times daily and cephalexin capsules 250 mg four times daily. Nonetheless, due to the lack of improvement, the patient was referred to Afzalipour Hospital, Kerman, for further assessment and management.

Laboratory testing revealed leukocytosis with a white blood cell count of 17 × 10^3^ per mm^3^ with a neutrophil predominance of 80%, normocytic anemia with hemoglobin of 9.7 g/dL, a mean corposcular volume of 79 fL and a platelet count of 435 × 10^3^ per mm^3^. The initial blood sample analysis indicated a C‐reactive protein level of 112 mg/dL and an erythrocyte sedimentation rate (ESR) of 44 mm/h.

**FIGURE 2:**
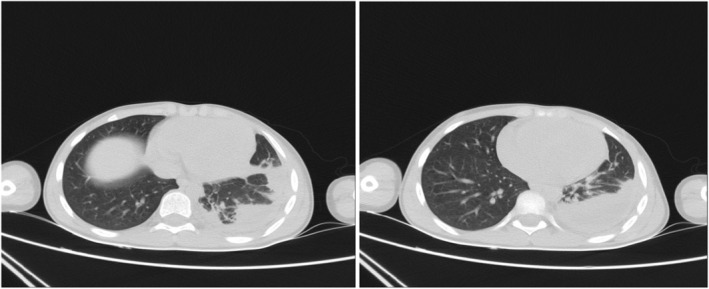
Spiral chest CT scan showing a left chest wall mass containing air bubbles and thickened septa in favor of empyema.

Based on the patient's history and physical examination, a chest radiograph was performed to evaluate the patient's clinical status. A radiological investigation revealed the existence of a mass‐like lesion in the left lower lobe. A chest CT scan was remarkable for left lower lobe cosolidation and loculated effusion, which contain air bubbles in favor of empyema (Figure 2).

## 3 METHODS

The patient underwent a sepsis workup, which included aerobic and anaerobic blood cultures, polymerase chain reaction (PCR) testing for respiratory viruses, and an analysis of sputum samples for Gram stain, acid‐fast bacilli (AFB), and pyogenic culture, which were found to be negative. In addition, a specimen of the purulent discharge from the patient's chest wall was collected and sent for Gram staining, AFB staining, and culture. Additionally, ultrasonography was conducted to assess the supraclavicular and axillary lymph nodes, indicating that their enlargement was attributed to reactive changes.

The pyogenic culture of the patient's discharge was positive for methicillin‐resistant *Staphylococcus aureus* (MRSA).

After confirming the diagnosis of EN, which happened as a complicated side effect of incompletely treated parapnuemonic effusion due to the lack of fibrinolytic injection in Zahedan Medical Center, the patient was started on a treatment plan involving intravenous (IV) administration of a broad‐spectrum antibiotic regimen, VATS, and finally a chest tube insertion. The empiric antibiotic regimen included vancomycin at a dosage of 200 mg four times daily, metronidazole at a dosage of 200 mg three times daily, cefepime at a dosage of 1 g three times daily, and meropenem at a dosage of 400 mg three times daily. To cover anaerobic bacteria, metronidazole was employed. For the coverage of gram‐negative bacteria, cefepime was administered. Meropenem was utilized to encompass a broad spectrum of activity, including both gram‐negative and gram‐positive bacteria, as well as anaerobic species. Vancomycin was employed specifically for covering MRSA. Following confirmation of empyema through culture results, which indicated MRSA, all antibiotic treatments were discontinued, and only intravenous vancomycin was maintained for a duration of 14 days, followed by a switch to oral vancomycin for an additional week according to the Infectious Diseases Society of America guidelines of 2011.^7^

As previously mentioned, the most important part of the treatment was VATS, which was identified as a crucial aspect of the treatment plan given the prolonged duration of the patient's EN. In the surgery performed for the patient, after making an incision along the midaxillary line, ports were inserted between the 4th and 5th intercostal spaces. Ports numbered 5 and 10 were implanted for the patient, and our surgeon entered the thoracic cavity under camera guidance. There were significant adhesions present in that area. These adhesions were meticulously dissected and thoroughly irrigated with ample amounts of saline solution. Additionally, necessary biopsies were taken from the pleural membrane and lung tissues to confirm the diagnosis by Gram staining and culture. The biopsies collected during VATS surgery were also sent for histopathological examination to provide detailed insights into the inflammatory changes, presence of pathogens, and tissue characteristics indicative of empyema.

After the surgery, a chest tube was inserted to facilitate the drainage of purulent secretions. It allowed for the continuous drainage of fluids from the affected area, thereby promoting the healing process and helping the patient recovery. Moreover, the fluid collected at the time of thoracotomy tube placement was sent for culturing, confirming the diagnosis.

The patient's postoperative course was closely monitored to ensure optimal recovery and resolution of symptoms. Following the VATS procedure and chest tube insertion, the patient's vital signs were monitored regularly, with particular attention given to temperature trends, respiratory rate, and oxygen saturation levels. Additionally, the drainage output from the chest tube was monitored to assess the volume and character of the fluid being evacuated. During the hospital stay, the patient received comprehensive care from a multidisciplinary team, including pulmonologists, infectious disease specialists, and thoracic surgeons. Nursing care included wound care for the chest tube insertion site, pain management, and respiratory support as needed.

The chest tube on the left side was successfully removed after a period of 7 days. In addition, an echocardiogram was conducted to assess for endocarditis, which yielded negative results. This approach is aimed at relieving pressure and reducing the risk of further complications associated with EN.

The patient's recovery was notably smooth and uneventful, with no reported complications. This positive outcome is a testament to the effectiveness of the treatment approach employed, which involved a combination of broad‐spectrum antibiotics, VATS, and the insertion of a chest tube for drainage. Overall, the patient responded well to the treatment plan and was able to make a full recovery without any significant issues.

After discharge from the hospital, regular outpatient clinic visits were scheduled to assess the patient's progress and response to treatment. During these visits, thorough physical examinations were conducted, including assessment of vital signs, respiratory status, and chest tube insertion site integrity. Close attention was given to the patient's respiratory symptoms, such as cough, dyspnea, and chest pain, to promptly detect any signs of recurrence of empyema.

The patient's antibiotic regimen was continued as prescribed, with regular reassessment of clinical response and potential adverse effects.

Overall, the follow‐up care after discharge from the hospital focused on continued monitoring, medical management, and patient education to support a successful recovery and minimize the risk of recurrence or complications associated with empyema.

## 4 CONCLUSION

In conclusion, EN is a rare but serious complication that can arise as a result of an underlying pneumonia or parapneumonic effusion. This case report highlights the importance of prompt recognition, diagnosis, and management of EN, particularly in pediatric patients. The case underscores the importance of a multidisciplinary approach to patient management, including close monitoring and follow‐up to prevent further complications. Increased awareness among healthcare professionals of the clinical features, diagnosis, and treatment options for EN would lead to early diagnosis and prompt intervention, ultimately improving patient outcomes.

## 5 DISCUSSION

EN is a rare and potentially life‐threatening condition that requires timely diagnosis and treatment to prevent significant morbidity and mortality.^8^, ^9^ Due to its uncommon nature, EN can be challenging to identify and manage.^1^, ^10^ EN occurs when an untreated empyema spreads through the parietal pleura and forms abscesses in the subcutaneous tissue of the anterior chest wall.^11^, ^12^ This often arises as a complication of an untreated parapneumonic effusion, which is the most common underlying cause and responsible for approximately 70% of cases.^8^

EN is typically caused by the same organism responsible for the pneumonia that preceded it.^2^, ^13^ According to a review of 26 cases published after 1966, *Mycobacterium tuberculosis* and *Actinomyces* species were found to be responsible for 75% (20/26) of cases.^14^ This highlights the importance of considering these organisms in the differential diagnosis of patients with suspected EN, particularly in areas with a high prevalence of tuberculosis. Accurate identification of the underlying organism is critical to guiding appropriate antimicrobial therapy and improving patient outcomes.

One of the less common causes of EN is *S. aureus*, with less than 10 case reports of EN caused by this bacterium found in a search of the PubMed database.

White‐Dzuro et al.^1^ reported a rare case of a 55‐year‐old male patient presenting with right shoulder and upper chest pain, diagnosed with methicillin‐sensitive *S. aureus* EN. As treatment, intravenous antibiotics were initiated, followed by surgical intervention and thoracic reconstruction to effectively manage the condition. In another study, Pugh^15^ reported a case of EN caused by MRSA in a 5‐year‐old boy with a ventricular shunt due to hydrocephalus and a history of type A influenza infection 1 week before the onset of fever and chest pain. The patient underwent treatment that included antibiotic therapy, chest tube insertion, and the infusion of a fibrinolytic agent to manage the condition. Moreover, Basndaru et al.^16^ documented a case of a 29‐year‐old man with a heroin addiction who presented with EN and a complex lesion on the tricuspid valve. The causative bacterial agent underlying these conditions was identified as *S. aureus* upon further evaluation.

In another study, DeSuza and Bush^17^ reported a case of a 70‐year‐old male patient with a past medical history of diabetes mellitus, hypertension, and diverticulitis, who presented with a 3‐week history of fever, night sweats, and right upper chest pain. The patient underwent appropriate clinical assessment, which revealed empyema necessitans (EN) due to methicillin‐sensitive *S. aureus*. Consequently, the patient received a 3‐week course of antibiotic treatment, accompanied by procedures such as pulmonary decortication via thoracotomy, evacuation of fluid from the chest wall, and debridement (Table 2).

**TABLE 2:** A summary of four case reports discussing EN due to *Staphylococcus aureus*.

No.	Title	First author (year)	Design	Age	Treatment	Duration of hospitalization	Outcome
1	Unusual presentation of empyema necessitans: case report and review of the literature	White‐Dzuro^1^ (2021)	Case report and review	55‐year‐old	Surgical management, antibiotic therapy (clindamycin)	Not mentioned	Discharged without any clinically evident residual infectious process with no complications except for moderate right shoulder stiffness that is managed with continuous physical therapy
2	Empyema necessitans a rare complication of methicillin‐resistant *Staphylococcus aureus* empyema in a child	Pugh^15^ (2020)	Case report	5‐year‐old	The shunt system was removed, admitted to the pediatric intensive critical care unit, chest tube insertion, fibrinolytic agent alteplase infusion, antibiotic therapy (vancomycin)	3 weeks	Discharged without any clinically evident residual infectious process
3	Empyema necessitans in the setting of methicillin‐susceptible *Staphylococcus aureus* causing pneumonia and bacteremia	Bandaru^16^ (2018)	Case report	29‐year‐old	Incision and drainage, chest tube insertion, antibiotic therapy (oxacillin)	6 weeks	The cavitary lesions throughout the lungs had resolved
4	Empyema necessitans due to methicillin‐sensitive *Staphylococcus aureus* case report and review	DeSuza^17^ (2020)	Case report and review	70‐year‐old	Thoracotomy, evacuation of fluid from the chest wall, debridement, chest tube insertion, antibiotic (ampicillin‐sulbactam)	1 month	Discharged without any clinically evident residual infectious process

In managing cases of loculated pleural empyema, the most effective approach is considered to be the injection of fibrinolytic agents into the pleural space, resulting in a complete recovery. If the designated timeframe for administering fibrinolytics for empyema treatment has elapsed, our therapeutic approach will transition to VATS with the option to switch to thoracotomy if required as a result of the chronic nature of the disease.^18^, ^19^, ^20^, ^21^ Conservative treatments such as thoracostomy tubes are often inadequate to manage organized empyemas, and studies have found that patients who undergo VATS have lower mortality rates and significantly reduced 30‐day readmission rates compared to those treated with tube thoracostomy.^22^ These findings highlight the importance of selecting the most appropriate treatment strategy for patients with loculated pleural empyema, as it can significantly impact their outcomes.

Alongside surgical interventions, the administration of appropriate antibiotics based on the microbial coverage determined from the patient's culture specimens plays a crucial role in the treatment process.

In the case discussed in this article, the patient did not receive treatment with fibrinolytic agents during their hospitalization at Zahedan Medical Center. As a result, the empyema treatment process was incomplete, leading to a disease relapse in EN. Due to the loss of the golden window for fibrinolytic injection in the patient, the patient was treated with VATS and chest tube insertion. This emphasizes the importance of personalized treatment plans and the need to consider all available options for patients with loculated pleural empyema, taking into account their unique circumstances and medical history.

## AUTHOR CONTRIBUTIONS

**Nazanin Zeinali Nezhad:** Conceptualization; data curation; investigation; resources; visualization; writing – original draft. **Aazam Gholami Shahrebabak:** Methodology; project administration; supervision; validation. **Amirhossein Shahpar:** Data curation; investigation; visualization; writing – review and editing.

## FUNDING INFORMATION

This research was conducted without external funding. All expenses associated with the research, including data collection, analysis, and publication, were covered by the authors personally. There was no financial support or sponsorship from any organization, institution, or funding agency for this study.

## CONFLICT OF INTEREST STATEMENT

The authors declare that they have no known conflict of financial interests or personal relationships that could have appeared to influence the work reported in this paper.

## ETHICAL APPROVAL

All procedures performed in studies involving human participants were in accordance with the ethical standards of the institutional and national research committee and with the 1964 Helsinki Declaration and its later amendments or comparable ethical standards. This article does not contain any studies with animals performed by any of the authors.

Every effort has been made to anonymize patient information to safeguard confidentiality. The patient's name, initials, and any other identifying information have been omitted from the report. Additionally, no identifiable images are included in this publication.

This case report encountered no ethical challenges during its preparation. The patient's autonomy and privacy were always respected, and their medical history and treatment were presented with utmost discretion and sensitivity.

The authors affirm their commitment to upholding ethical principles in medical publishing, prioritizing patient welfare, confidentiality, and informed consent throughout the reporting process.

## CONSENT

Written informed consent was obtained from the patient to publish this report in accordance with the journal's patient consent policy.

## Data Availability

The data that support the findings of this study are available from the corresponding author upon reasonable request.
